# Panoramic Visual SLAM Technology for Spherical Images

**DOI:** 10.3390/s21030705

**Published:** 2021-01-21

**Authors:** Yi Zhang, Fei Huang

**Affiliations:** School of Geodesy and Geomatics, Wuhan University, Wuhan 430079, China; 13277066582@163.com

**Keywords:** panoramic vision, SLAM, spherical imaging model, SPHORB, ternary SIFT

## Abstract

Simultaneous Localization and Mapping (SLAM) technology is one of the best methods for fast 3D reconstruction and mapping. However, the accuracy of SLAM is not always high enough, which is currently the subject of much research interest. Panoramic vision can provide us with a wide range of angles of view, many feature points, and rich information. The panoramic multi-view cross-imaging feature can be used to realize instantaneous omnidirectional spatial information acquisition and improve the positioning accuracy of SLAM. In this study, we investigated panoramic visual SLAM positioning technology, including three core research points: (1) the spherical imaging model; (2) spherical image feature extraction and matching methods, including the Spherical Oriented FAST and Rotated BRIEF (SPHORB) and ternary scale-invariant feature transform (SIFT) algorithms; and (3) the panoramic visual SLAM algorithm. The experimental results show that the method of panoramic visual SLAM can improve the robustness and accuracy of a SLAM system.

## 1. Introduction

Simultaneous Localization and Mapping (SLAM) is an advanced technology in the area of robot navigation, pilotless driving, unmanned aerial vehicle surveying and mapping, and virtual reality (VR)/augmented reality (AR). It refers to the use of a sensor in an unfamiliar environment, where the data observed by the sensor are used to estimate the state of motion of the sensor itself, while building a map of the surrounding environment. SLAM technology can be divided into LiDAR SLAM and visual SLAM. For historical reasons, the research into LiDAR SLAM began earlier than research into visual SLAM, and LiDAR SLAM technology is more mature than visual SLAM technology in theory, algorithms, and landing products. However, LiDAR is more expensive than cameras, and LiDAR has a limited range of detection. Cameras have no distance limit and cost less. At present, the solutions for visual SLAM technology are mainly based on RGB-D cameras and monocular, stereo, or panoramic cameras. The biggest difference between the two schemes is that RGB-D cameras are equipped with depth sensors, whereas ordinary monocular, stereo, and panoramic cameras are not. Since RGB-D cameras are generally more expensive than ordinary cameras, it is of great significance to study visual SLAM technology based on ordinary cameras (like monocular, stereo, or panoramic cameras without depth sensors), to reduce the cost. Among the ordinary cameras, panoramic cameras have gradually become one of the hotspots in the field of visual SLAM research because of their wide range of information perception and fast and complete information acquisition.

The common monocular camera has a horizontal angle of view of about 60 degrees and a vertical angle of view of about 45 degrees. When a mobile platform moves continuously, because of the small field of vision, the extracted feature points only stay in the field of vision for a short period of time. As a result, mobile platforms cannot observe the feature points continuously and effectively, which limits the development of SLAM based on visual sensors. The longer the continuous observation time of the feature points, the more conducive this is to system state correction and updating [1]. Davison and Murray [2] also noted that the longer the time of continuous feature observation, the faster the error convergence, and the shorter the time of continuous feature observation, the more difficult it is to effectively reduce the system uncertainty and positioning error. Therefore, compared with a limited FOV camera, using a panoramic camera with a full view is a better way.

## 2. Related Works

The earliest real-time monocular visual SLAM system was MonoSLAM [3], proposed by Professor Davison in 2007. The system uses an extended Kalman filter (EKF) as the back-end and tracks very sparse feature points on the front-end. It is considered the birthplace of a lot of work. In the same year, Klein and others proposed the PTAM [4]. This system realizes the parallelization of tracking and mapping. It is the first solution to use nonlinear optimization instead of using traditional filters as the back-end. This solution is also the main solution for later SLAM systems. The Oriented FAST and Rotated BRIEF (ORB)-SLAM [5,6] proposed by Mur-Artal et al. inherited the advantages of PTAM. It extended the two-thread structure to a three-thread structure (tracking, mapping, loop detection), and achieved good tracking and mapping effects. ORB-SLAM [5,6] is the peak of SLAM based on the feature point method. Many systems use it as the basic framework, such as PL-SLAM [7,8], fisheye-SLAM [9], multicol-SLAM [10], and so on. This paper is also based on its framework and expands the imaging model of panoramic spherical images. The above mentioned is SLAM based on the feature point method (also known as the indirect method). The opposite of the indirect method is the direct method. This method does not need to extract the feature points of the image, and directly tracks the optical flow on the image. Representative works include large-scale direct monocular SLAM (LSD-SLAM) [11] and direct sparse odometry (DSO) [12]. Compared with the indirect method, the direct method has advantages in weak texture areas. However, it also has disadvantages, such as sensitivity to camera parameters and exposure, and it cannot be used in wide-baseline shooting modes or scenes with large viewing angle changes. The fast semi-direct monocular visual odometry (SVO) [13] proposed by Forster et al. in 2014 is a visual odometry based on the sparse direct method (also known as the semi-direct method), which uses a mixture of feature points and the direct method. It neither requires calculating descriptors nor processing the massive information of dense or semi-dense maps, so the processing speed of SVO is extremely fast. However, it is mainly for the top view scene of the UAV platform, and there is no back-end optimization and loop detection part.

Due to the shortcomings in the narrow viewing angle of monocular cameras, more and more research has tended to use fisheye and panoramic cameras with larger viewing angles to achieve a more robust SLAM system. In 2007, Kangni et al. [14] proposed a panoramic image pose calculation method. It uses paired basic matrices to restore the position of the panoramic camera in the scene, and optimizes the pose through bundle adjustment [15]. The articles [16,17,18,19] propose better methods for some theories of panoramic images (such as calibration, synthesis, imaging models, etc.). These methods lay a good foundation for the researchers in this field. In 2010, Rituerto et al. [20] implemented a panoramic vision SLAM system based on the EKF algorithm, and verified that its positioning accuracy is better than monocular SLAM. In 2011, Gutierrez et al. [21] introduced a new computation of the descriptor patch for catadioptric omnidirectional cameras that aimed to reach rotation and scale invariance. In 2015, Gamallo et al. [22] proposed a SLAM algorithm (OV-FastSLAM) for omnidirectional cameras operating with severe occlusions. The three works of Valiente et al. [23,24,25] are all related to panoramic visual SLAM and based on the EKF algorithm. Li et al. [26] presented a SLAM system based on a spherical model for full-view images in indoor environments in 2018. Seok et al. [27] presented robust omnidirectional visual odometry for wide-baseline wide-FOV camera systems (ROVO) in 2019. The hybrid projection model in their paper combines the perspective and cylindrical projection to maximize the overlap between views and minimize the image distortion that degrades feature matching performance. There are also some excellent open-source works (or studies based on open-source works) that relate to fisheye or panoramic visual SLAM. For example, fisheye-SLAM [9], ORB-SLAM3 [28,29], and PAN-SLAM [30] are based on ORB-SLAM2 [6]. Fisheye-SLAM [9] and ORB-SLAM3 [28,29] implement fisheye visual SLAM. PAN-SLAM [30] implements a panoramic visual SLAM based on a multi-camera system. Caruso et al. [31] proposed large-scale direct SLAM for omnidirectional cameras based on LSD-SLAM. Liu et al. [32] and Matsuki et al. [33] respectively proposed fisheye-stereo DSO and omnidirectional DSO based on DSO [12]. Forster et al. [34] and Heng et al. [35], respectively, proposed multi-camera system SVO and fisheye-stereo SVO based on SVO [13]. OpenVSLAM [36] implements a versatile visual SLAM framework with high usability and extensibility. The system can deal with various types of camera models, such as perspective, fisheye, and equirectangular.

The main aim of this work is to make full use of the omnidirectional perspective of panoramic vision and SLAM technology to achieve a higher positioning accuracy than monocular visual SLAM, while focusing on the spherical imaging model and the problems in feature extraction and matching. The main contributions of this paper are as follows.

(1) The panoramic imaging model. We study the pixel expression method for spherical images, and derive the formula between the pixel coordinates and camera coordinates.
(2) Feature extraction and matching of panoramic images. Because panoramic images are seriously distorted and the imaging model differs from that of an ordinary monocular camera, we compare and analyze the feature extraction effects of various algorithms. The Spherical Oriented FAST and Rotated BRIEF (SPHORB) feature extraction algorithm is identified as being the most suitable for a panoramic visual SLAM positioning system. In addition, we propose improvements to the scale-invariant feature transform (SIFT) algorithm, and realize binary SIFT and ternary SIFT. These improvements to SIFT greatly increase the speed of SIFT while ensuring sufficient accuracy.
(3) Research into a SLAM algorithm for panoramic vision and the implementation of a location system. The ORB-SLAM2 [6] algorithm is improved, via front-end odometry and back-end optimization, to realize a SLAM positioning system that is suitable for panoramic vision.

## 3. Overview of Our Method

Mobile panoramic visual imaging mainly adopts three modes: multi-lens combination, rotation, and refraction [16,37]. The current mainstream approach is to capture panoramas through multi-lens combinations, such as Point Grey’s Ladybug series. It consists of six fisheye lenses with very high resolution, but it is too expensive and this has reduced its popularity. Ricoh is a consumer-grade panoramic camera. It is composed of two fisheye lenses, which are sufficient for the experimental resolution of this paper.The experimental data in this paper include two parts: the simulation data of the InteriorNet dataset [38] and the measured data collected by a Ricoh camera.

**Figure 1:**
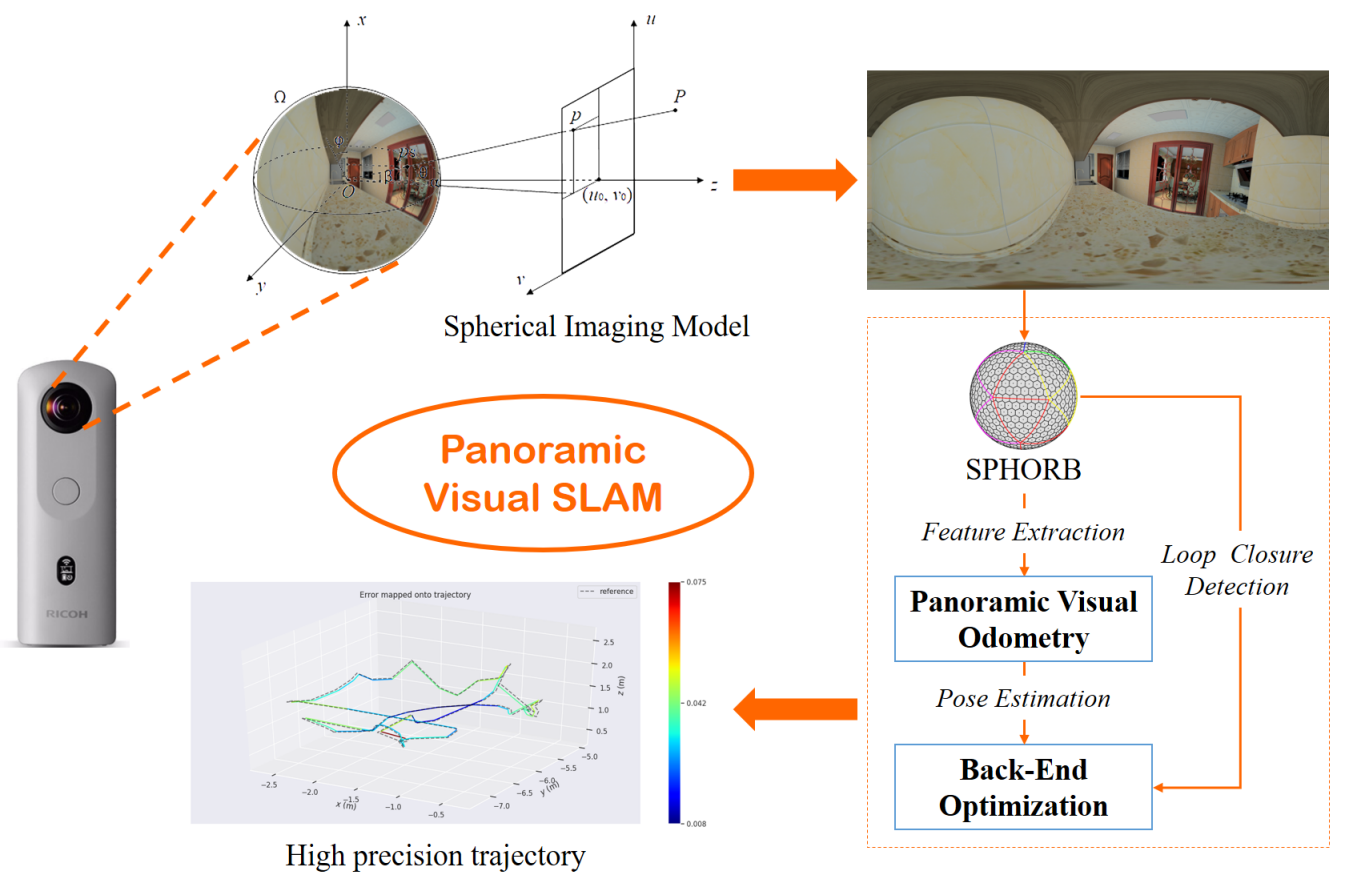
Flow chart of panoramic visual Simultaneous Localization and Mapping (SLAM).

This paper proposes a SLAM method based on panoramic vision and its overall flow chart is shown in Figure 1. Our system is based on ORB-SLAM2 [6] for development and improvement. We extended it for a spherical image. Firstly, the collected data are transformed by the spherical imaging model (please refer to Section 4) to synthesize a 360-degree panoramic image. The SPHORB algorithm is then used as the front-end of the SLAM system to extract features of panoramic images and realize panoramic visual odometry. Next, the position and pose of the panoramic camera are optimized at the back-end with g2o [39]. Loop closure detection is carried out at the same time. The experimental results show that the proposed method is more efficient, accurate, and robust than monocular vision for pose estimation.

## 4. The Spherical Imaging Model

Unlike monocular cameras, the image distortion of fisheye and panoramic cameras is very serious. The traditional perspective model is no longer applicable. Many researchers have proposed unique models based on the imaging principles of fisheye and panoramic cameras, while other researchers have proposed imaging models that can describe perspective, fisheye, and panoramic images with a unified model. In 2000, Geyer et al. provided a unified theory for all central catadioptric systems [40]. This model was extended by Barreto et al. in 2001, which was known as the spherical camera model [41]. It can model central catadioptric systems and conventional cameras. In 2015, Khomutenko et al. further extended the model and proposed an enhanced unified camera model (EUCM) [42]. The new model applies to catadioptric systems and wide-angle fisheye cameras. It does not require additional mapping to model distortions, and it takes just two of the projection parameters using a simple pinhole model to represent radial distortion. This model was used in fisheye-SLAM [9] and achieved good results. In 2018, Usenko et al. propose the double sphere camera model [43], which fits well with large field-of-view lenses. It is computationally friendly and has a closed-form inverse.

**Figure 2:**
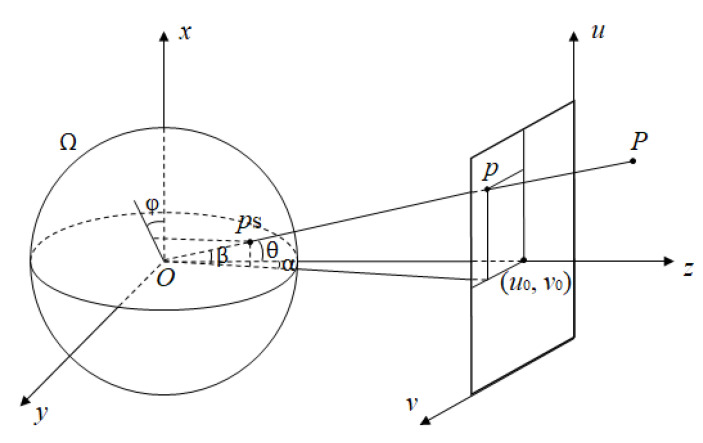
Spherical imaging diagram.

In this paper, we use a spherical imaging model called “longitude and latitude expression”. This method avoids complicated description parameters. It compares the panoramic spherical image to the Earth, and the pixel coordinates to the latitude and longitude. As shown in Figure 2, O−xyz is the camera coordinate system. The pixel coordinates of the projection points of object point $P(X_{w},Y_{w},Z_{w})$ in a planar image are p(u,v). The projection point on the spherical image is $p_{s}$, which can be expressed in latitude and longitude as $p_{s}\left(\theta ,\varphi \right)$.

P(X,Y,Z) is the object point, which is equivalent to the $P(X_{w},Y_{w},Z_{w})$ mentioned above. $p_{s}\left(x,y,z\right)$ is the corresponding projection point on the sphere. p(u,v) is the corresponding point on the plane.

We let α be the angle between the projection of vector $\vec{Op_{s}}$ on plane O−yz and the *z*-axis, and β be the angle between vector $\vec{Op_{s}}$ and plane O−yz. In real images, *u* and *v* correspond to the rows and columns of the image pixels, respectively, which are finite values. So $\alpha ,\beta \in [-\frac{\pi }{2},\frac{\pi }{2}]$. According to the spatial geometric relations, a formula can be derived:

(1) $$\left\{\begin{array}{cc} \alpha & =\arctan\frac{v-v_{0}}{f}\\ \beta & =\arctan\frac{u-u_{0}}{\sqrt{f^{2}+{\left(v-v_{0}\right)}^{2}}} \end{array}\right.$$

where *f* is the focal length of the camera, and $\left(u_{0},v_{0}\right)$ are the pixel coordinates of the principal point. Equation (1) expresses the mapping relationship between the panoramic planar image and the panoramic spherical image.

Panoramic spherical image means that the panoramic image acquired by the camera is mapped to a virtual spherical surface in space, which emphasizes the imaging process of the image. Panoramic planar image is the image output by the camera, which is similar to the planar image we see on paper, and emphasizes the appearance of the image in front of us. In this paper, spherical images refer to panoramic spherical images, and panoramic images refer to panoramic planar images. Generally speaking, there is no difference, but the emphasis is different.

**Figure 3:**
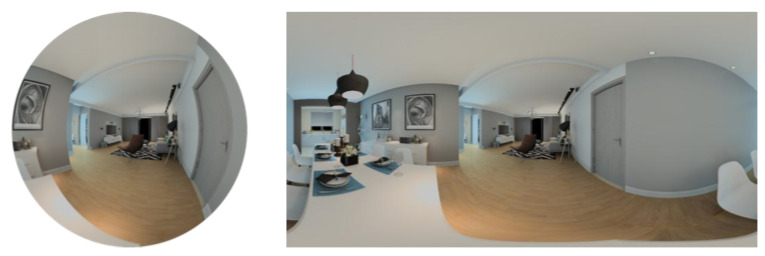
Panoramic spherical image and panoramic planar image.

When the spherical image is mapped to the plane completely, the aspect ratio of the planar image must be 2:1 (see Figure 3). The mapping relationship between the planar image and spherical image is just like that between a map and the Earth. The latitude and longitude (θ,φ) in the spherical image correspond to the rows and columns (u,v) of the planar image. The latitude θ∈[0,π] is divided into *H* number of equivalents, corresponding to row u∈[0,H] of the planar image. The longitude φ∈[0,2π] is divided into *W* number of equivalents, corresponding to column v∈[0,W] of the planar image. In this way, the spherical image is mapped to a planar image with a resolution of W×H. According to this, we can construct a two-dimensional array to express the spherical pixels.

According to Equation (1), $p_{s}$ can be expressed as $p_{s}\left(\alpha ,\beta \right)$. Therefore, $p_{s}\left(\alpha ,\beta \right)$ can be used to express p(u,v) and $p_{s}\left(x,y,z\right)$ (see Equations (2) and (3)). In the formulas, *W* and *H* respectively represent the width and height of the panoramic image.

(2) $$\left\{\begin{array}{cc} u & =r(\alpha +\pi )\\ v & =r(\frac{\pi }{2}-\beta )\\ r & =\frac{W}{2\pi }=\frac{H}{\pi } \end{array}\right.$$

(3) $$\left\{\begin{array}{cc} x & =r\cos\beta \cos(\frac{\pi }{2}-\alpha )\\ y & =r\cos\beta \sin(\frac{\pi }{2}-\alpha )\\ z & =r\sin\beta \end{array}\right.$$

We let $P_{c}\left(X_{c},Y_{c},Z_{c}\right)$ be the camera coordinates of P(X,Y,Z). Because the optical center *O* and spherical projection points $p_{s}$ and $P_{c}$ are collinear, Equation (4) can be obtained, and Equation (5) is then established, where *R* is the distance between the object square point and the optical center of the camera.

(4) $$\frac{X_{c}}{x}=\frac{Y_{c}}{y}=\frac{Z_{c}}{z}$$

(5) $$X_{c}^{2}+Y_{c}^{2}+Z_{c}^{2}=R^{2}$$

According to Equations (2) and (3), the relationship between the panoramic spherical coordinates and the pixel coordinates can be derived, as shown in Equations (6) and (7).

(6) $$\left\{\begin{array}{cc} x & =r\cos\left(\frac{\pi }{2}-\pi \frac{v}{H}\right)\cos\left(\frac{3\pi }{2}-2\pi \frac{u}{W}\right)\\ y & =r\cos\left(\frac{\pi }{2}-\pi \frac{v}{H}\right)\sin\left(\frac{3\pi }{2}-2\pi \frac{u}{W}\right)\\ z & =r\sin(\frac{\pi }{2}-\pi \frac{v}{H}) \end{array}\right.$$

(7) $$\left\{\begin{array}{cc} u & =\frac{3W}{4}-\frac{W}{2\pi }\arctan\frac{y}{x}\\ v & =\frac{H}{2}-\frac{H}{\pi }\arcsin\frac{z}{r} \end{array}\right.$$

By combining Equations (4) and (7), the relationship between pixel coordinates p(u,v) and camera coordinates $P_{c}\left(X_{c},Y_{c},Z_{c}\right)$ can be derived, as shown in Equation (8).

(8) $$\left\{\begin{array}{cc} u & =\frac{3W}{4}-\frac{W}{2\pi }\arctan\frac{Y_{c}}{X_{c}}\\ v & =\frac{H}{2}-\frac{H}{\pi }\arcsin\frac{Z_{c}}{\sqrt{X_{c}^{2}+Y_{c}^{2}+Z_{c}^{2}}} \end{array}\right.$$

## 5. Feature Extraction and Matching of Spherical Images

A few feature extraction algorithms have been designed for use with spherical images, such as spherical SIFT [44], PCA-SIFT [45], etc. Although, to a certain extent, the influence of spherical image distortion on feature extraction is solved, the speed of the feature extraction is not ideal. The main concern of this paper is panoramic visual SLAM positioning technology, which requires the system to output the real-time pose information of the camera. Therefore, the algorithms with poor real-time performance are not discussed.

In a real-time visual SLAM system, in order to ensure that the speed of the feature extraction matches that of the system, it is usually necessary to reduce the quality of the feature extraction. One solution for monocular vision SLAM systems is to use the Oriented FAST and Rotated BRIEF (ORB) algorithm [46] to complete the feature extraction and matching. However, in panoramic vision, because of the influence of the image distortion, and the fact that the camera imaging model differs from that of monocular vision, the ORB algorithm is not ideal for the feature extraction of panoramic images.

**Figure 4:**
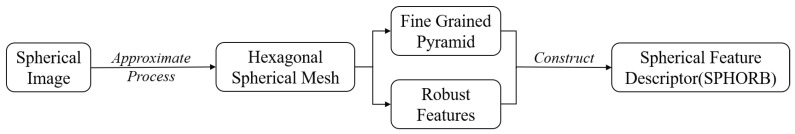
Spherical Oriented FAST and Rotated BRIEF (SPHORB) algorithm flow chart.

The SPHORB algorithm stems from the geodesic grid and can be considered as an equal-area hexagonal grid parametrization of the sphere used in climate modeling. It has been proved in topology that any surface can be approximated by triangulation. Therefore, a sphere can also be approximated by triangles, which can be combined into hexagonal meshes (and may contain a small number of pentagons). The idea of the SPHORB algorithm is to approximate the spherical image and obtain a hexagonal spherical mesh (similar to a football). The fine-grained and robust features are then directly constructed on the hexagonal spherical grid, avoiding the time-consuming computation of spherical harmonics and the related bandwidth constraints, thus enabling a very fast performance and high descriptive quality (the specific process is shown in Figure 4). We therefore use the SPHORB algorithm for the feature extraction.

## 6. The Panoramic Visual SLAM Algorithm

The SLAM problem can be described by two equations: the motion equation (Equation (9)) and the observation equation (Equation (10)).

(9) $$x_{k}=f\left(x_{k-1},u_{k},w_{k}\right)$$

(10) $$z_{k,j}=h\left(y_{j},x_{k},v_{k,j}\right)$$

In the motion equation, subscript *k* denotes the current time serial number, and k−1 denotes the last moment. $u_{k}$ is the sensor’s reading and $w_{k}$ is the noise. $x_{k}$ represents the position of the sensor at the current time. $x_{k}$ is a three-dimensional vector. $x_{k-1}$ represents the position of the sensor at the last moment.

In the observation equation, subscript *j* represents the ordinal number of the currently observed landmarks. $y_{j}$ is the landmark observed by the sensor at position $x_{k}$, which is also a three-dimensional vector. $z_{k,j}$ denotes the observation data corresponding to the landmarks $y_{j}$. $v_{k,j}$ is the measurement noise.

These two equations are the most basic equations in the SLAM problem. They describe the motion and observation models of the sensor in the SLAM problem. Therefore, the problem can be abstracted as follows: how to solve the location problem (estimate *x*) and the mapping problem (estimate *y*) when we know the reading data of the motion measurement and the reading data of the sensor. At this time, we model the SLAM problem as a state estimation problem, i.e., how to estimate the internal and hidden state variables by measuring data with noise [47].

In this paper, we mainly address the location problem of panoramic SLAM, i.e., how to solve the x-vector in the above-mentioned state estimation problem, the position and attitude of the panoramic camera, and how to make full use of the wide-range perspective of the panoramic camera to optimize the vector *x*.

**Figure 5:**
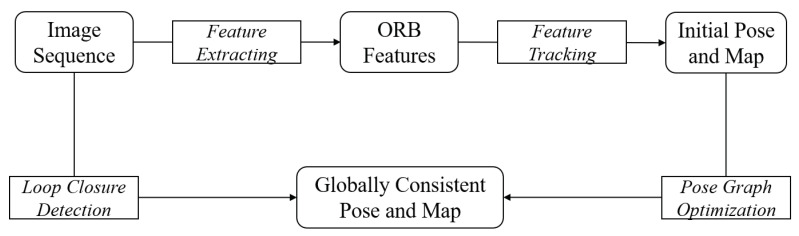
Framework of classical visual SLAM algorithms.

The algorithm framework of classical visual SLAM is shown in Figure 5. Firstly, the data of the visual sensor, including the video and image data, are input. Secondly, feature extraction and matching of the image data are carried out. The transform matrix T (including rotation matrix R and translation vector *t*) is calculated according to the principle of reprojection error minimization, and the pose change of the camera is estimated. At the same time, a local map and the initial pose map are constructed. Next, in the back-end optimization, considering the loop information, the transformation matrix T and the three-dimensional coordinate *X* of the landmark are optimized simultaneously by using the non-linear optimization method. Finally, sparse three-dimensional point clouds are generated.

### 6.1. Front-End Visual Odometry

Compared with the classical SLAM algorithm framework, the SLAM algorithm based on panoramic vision faces some problems: (1) the distortion of the spherical image makes the feature extraction and matching difficult; (2) the mapping relationship between the pixel coordinates and camera coordinates of the planar image is not applicable to a spherical surface; and (3) the method of solving pose with a polar constraint of the planar image is not applicable to a spherical surface.

**Figure 6:**
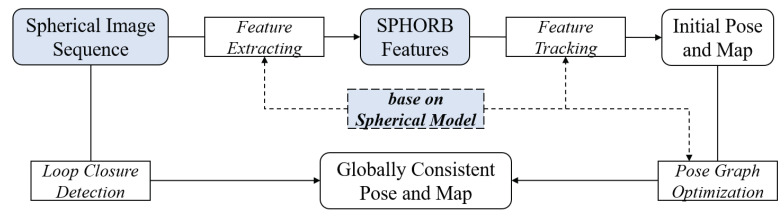
Framework of panoramic visual SLAM algorithms. Improvements to the classical framework are shown in blue.

Therefore, in view of the panoramic visual SLAM positioning problem, we need to improve the front-end visual odometry part of the classical visual SLAM framework. The improvement process is shown in Figure 6. To deal with the distortion of spherical images, the SPHORB algorithm, which can directly extract and match the features of a spherical surface, is adopted to effectively reduce the influence of image distortion on feature extraction and matching. From the pixel coordinates to the camera coordinates, the planar image is described by an internal reference matrix, while the panoramic image is a sphere. The mapping relationship between the pixel coordinates (u,v) and camera coordinates (θ,φ) needs to be described by a latitude and longitude expression.

### 6.2. Back-End Optimization

Since the polar geometric relationship of a spherical panorama is consistent with that of a planar image, the essential matrix E between two spherical coordinate systems can be calculated directly using the coordinates of standard spherical panoramic image points. Therefore, the polar-constrained relationship of the planar image $x_{2}^{T}Ex_{1}=0$ can be extended to the sphere. $x_{1},x_{2}$ are the panoramic spherical coordinates $\left(x_{1},y_{1},z_{1}\right),\left(x_{2},y_{2},z_{2}\right)$, which represent a pair of namesake points $p_{1},p_{2}$. The panoramic spherical coordinates can be directly calculated by Equations (6) and (7).

In this study, the back-end optimization algorithm in ORB-SLAM2 [6] was improved to enable it to handle the spherical model. In the optimization process of the back-end of the sphere, we still use the pixel reprojection error, and the error function can be expressed as shown in Equation (11). $x_{\hat{p}}$ is the pixel coordinate of the point after reprojection, and $x_{p}$ is the pixel coordinate of the matching point.

(11) $$e=\frac{1}{2}\sum_{i=1}^{n}\left|\right|x_{\hat{p}}-x_{p}{\left|\right|}_{2}^{2}$$

In order to optimize the overall reprojection error, the least-squares problem is constructed. All the positions are adjusted to minimize *e*. By combining Equations (8) and (11), the Jacobian matrix of the reprojection error point $P_{c}\left(X_{c},Y_{c},Z_{c}\right)$ can be obtained as shown in Equation (12). The Jacobian matrix of pose ξ is shown in Equation (13). 

We let $R=\sqrt{X_{c}^{2}+Y_{c}^{2}+Z_{c}^{2}},a=\sqrt{X_{c}^{2}+Y_{c}^{2}}$.

(12) $$J=\frac{\partial e}{\partial P_{c}}=-\left[\begin{array}{ccc} \;\frac{WY_{c}}{2\pi a^{2}} & -\frac{WX_{c}}{2\pi a^{2}} & 0\\ \frac{HZ_{c}X_{c}}{\pi R^{2}a} & \frac{HZ_{c}Y_{c}}{\pi R^{2}a} & -\frac{Ha}{\pi R^{2}} \end{array}\right]$$

(13) $$J=\frac{\partial e}{\partial \xi }=-\left[\begin{array}{cccccc} \;\frac{WY_{c}}{2\pi a^{2}} & -\frac{WX_{c}}{2\pi a^{2}} & 0 & \frac{WZ_{c}X_{c}}{2\pi a^{2}} & -\frac{WZ_{c}Y_{c}}{2\pi a^{2}} & -\frac{W}{2\pi }\\ \frac{HZ_{c}X_{c}}{\pi R^{2}a} & \frac{HZ_{c}Y_{c}}{\pi R^{2}a} & -\frac{Ha}{\pi R^{2}} & -\frac{HY_{c}}{\pi a} & \frac{HX_{c}}{\pi a} & 0 \end{array}\right]$$

where *e* represents the reprojection error, $P_{c}$ represents the camera coordinates of the object points, and ξ represents the Lie-algebraic form of the pose.

So far, we have derived the Jacobian matrix of the observation equation of the panoramic camera from the camera pose and feature points, which are an important part of the back-end optimization. They are also the unique part that distinguishes a panoramic camera from a monocular camera in the process of back-end optimization.

## 7. Experiments and Analysis

### 7.1. Experimental Data

**Figure 7:**
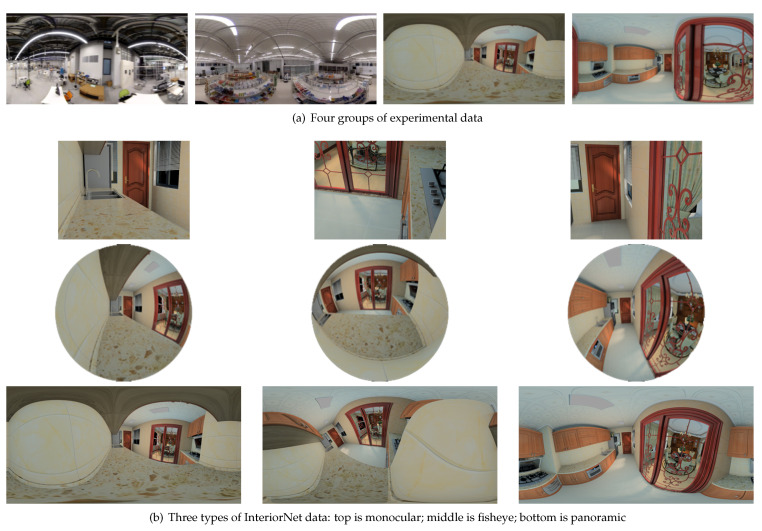
Experimental image data.

In order to test the robustness and accuracy of panoramic visual SLAM in different environments, four datasets were selected (see Figure 7a). The first two groups were from our measured data, while the latter two groups were from InteriorNet data. The trajectory of our measured data was roughly a rectangle, and the movement of the camera was relatively stable. InteriorNet data were simulated by a computer. It could arbitrarily change the viewpoint to generate a panoramic image, so its trajectory was irregular. We used these two different types of data to evaluate the robustness of the algorithm. The InteriorNet data were generated by Li et al. [38] in a simulated environment. Each InteriorNet dataset contains panoramic data, plus corresponding monocular data and fisheye data (as shown in Figure 7b), each with 1000 frames of images. The movement of the measured data was relatively stable, while the data generated in the simulated environment showed more violent movement. In this paper, the robustness and accuracy of panoramic visual SLAM and monocular visual SLAM are evaluated through the data of various scenes and motion states.

### 7.2. Matching Experiment

#### 7.2.1. SIFT, Binary SIFT, and Ternary SIFT

Because SIFT has good robustness to scale and rotation, and its accuracy is high but its speed is slow, we attempted to improve its speed so that it could be used in SLAM. The main improvement was to quantize the 128-dimensional floating-point vector (128×32=4096 bits) of SIFT with the median as the bound, and to binarize the original floating-point numbers. The numbers greater than the median were recoded to 1, and the numbers less than the median were recoded to 0, so that the data were compressed into 128 bits. This can greatly reduce the memory consumption and improve the matching speed, while maintaining the robustness of SIFT.

Similarly, in order to quantify the original 128-dimensional floating-point vector more accurately, we implemented “ternary” SIFT. At the same time, taking the values at 1/4 and the median as the boundaries, the encoding from small to large was 00, 10, and 11. The original 32-bit floating-point numbers were compressed into 2 bits, with a total of 256 bits.

**Figure 8:**
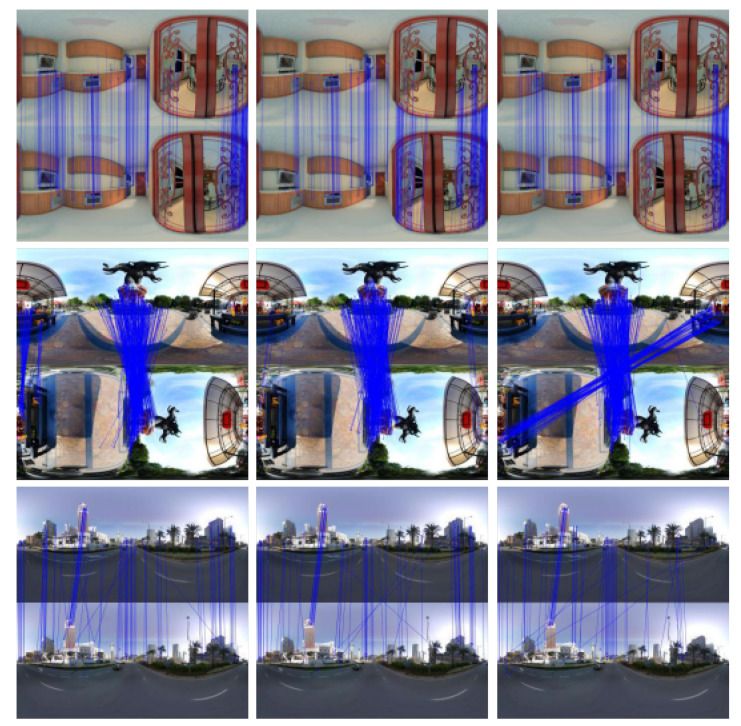
(**left**) sift; (**middle**) binary scale-invariant feature transform (SIFT); (**right**) ternary SIFT.

In the experiments, because the parts of feature extraction and descriptor calculation were the same, the time taken for the quantization descriptor could be ignored, so that the matching speed and accuracy of the three methods could be compared. The coarse matching was screened by a ratio test, for which the threshold was 0.8. The fundamental matrix was calculated by random sample consensus (RANSAC), and a reprojection error of 3 pixels was used for the fine matching. After several groups of experiments, three pairs of typical panoramic images were selected for analysis. The first pair was made up of indoor images with more feature points, without too large a rotation angle, which is a common situation in SLAM. The second pair was made up of images with a 90 degree rotation. The third group was made up of outdoor images with fewer feature points. The experimental results are shown in Figure 8. The left, middle, and right are the results of SIFT, binary SIFT, and ternary SIFT, respectively.

The evaluation of matching results for different kinds of SIFT are listed in Table 1. For the case of more feature points, as in the first group of data, the matching data and fine matching rate of the three methods are almost the same, but the speed of SIFT is significantly slower than that of binary SIFT and ternary SIFT. The matching result of ternary SIFT is better, and even better than SIFT in the case of rotation, and the speed is also faster, as shown in the second group of data. For the case of fewer feature points, the matching results of binary SIFT and ternary SIFT are worse than that of SIFT. The reason for this may be that the number of matching points is small, and the proportion of wrong matches in the coarse matching is high, which leads to the increase of iterations in RANSAC.

In general, the matching speed of ternary SIFT is the fastest. In the case of more feature points, a superior matching result can be obtained, even if the image has rotation.

#### 7.2.2. SPHORB and ORB

The ORB algorithm is one of the fastest feature extraction algorithms available, and has good matching accuracy, but it is mainly used for processing planar images. For spherical images, the ORB algorithm does not work as well. The SPHORB algorithm is a feature extraction algorithm used to process spherical images, and is an improvement of the ORB algorithm based on the features of a spherical image (please refer to Section 5), ensuring faster processing speed and higher accuracy.

**Figure 9:**
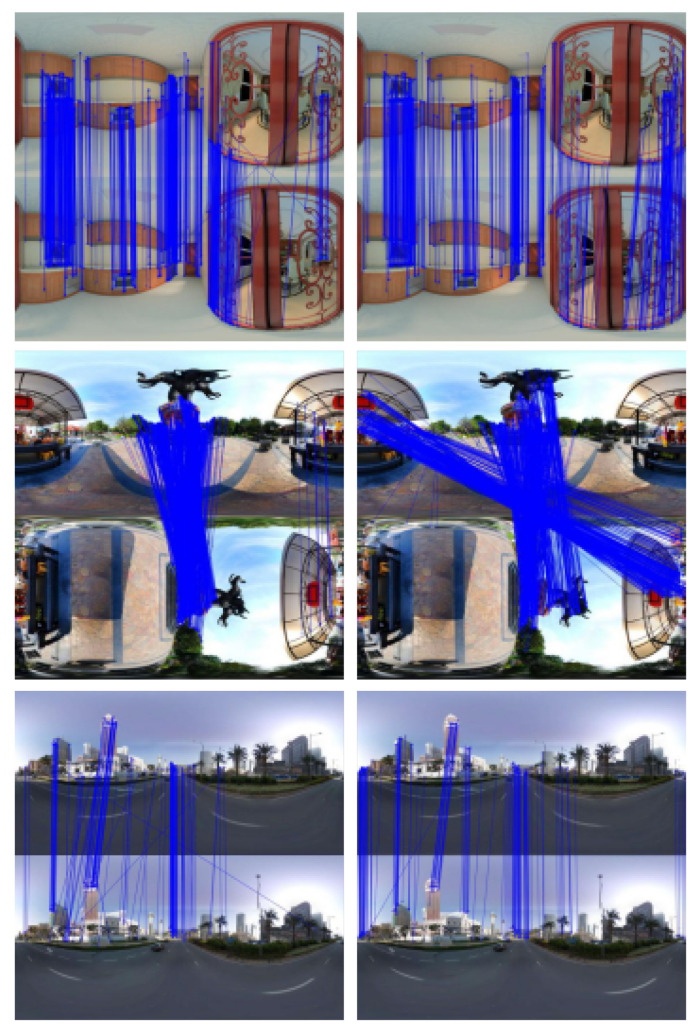
(**left**) Oriented FAST and Rotated BRIEF (ORB); (**right**) SPHORB.

In the panoramic image-matching experiments, the three datasets described in Section 7.2.1 were again used. The feature points calculated by ORB and SPHORB were used for the matching in the three experiments. Figure 9 shows the matching result of the ORB algorithm on the left and the SPHORB algorithm on the right.

As shown in Figure 9, in the first and third groups of experiments, the matching lines of the SPHORB have better consistency and fewer crossover lines. The figure shows that the matching quality was better than ORB. In the second experiment, because the image was rotated 90 degrees, the ORB algorithm only matched the central part of the image, but the feature with the same name on the edge was not matched. However, the SPHORB algorithm could match most of the eponymous feature points in both the center and the edge.

The evaluation of matching results for ORB and SPHORB are listed in Table 2. The filtering rules for the rough matching and fine matching are consistent with those described in Section 7.2.1. However, the results from the first and second sets of data experiments showed that the ORB algorithm had a higher matching precision than the SPHORB algorithm. Notably, in the second set, SPHORB had a fine matching rate of only 24.86%, which is clearly not true. The reason for this is most likely the removal of a large number of correct matches during the RANSAC process. As described in Section 7.2.1, the RANSAC algorithm in OpenCV was adopted, which is mainly used for planar images. For panoramic images, the effect of removing mismatches is often not good, especially when a pair of panoramic images has a large rotation angle (as in the second group of data). Therefore, a special spherical RANSAC method is needed to obtain a reliable and precise matching rate. This will be addressed in our future research.

In summary, the matching results show that the accuracy the SPHORB algorithm is higher than the ORB algorithm. Because the current filtering rules for the precise matching of spherical images are unreliable, the data results in Table 2 do not reflect the true accuracy of SPHORB.

### 7.3. Panoramic Visual SLAM Experiment

According to the data characteristics, the experiments were divided into two groups. The first group of data was made up of measured data without the true values of the trajectories. These data were used to evaluate the mapping effects of ORB and SPHORB in SLAM, including the initialization speed, the number of matches per frame, and the tracking time of each frame. The initialization speed was measured by the ID of the frame where the initialization was successful. We recorded the number of successful matching points in each frame and calculated their mean value. The greater the number of matching points, the better the accuracy of SLAM. Finally, the average tracking speed in each frame were recorded. The second group of data was made up of the InteriorNet simulation data, and because the data provided the true values of the trajectories, they could be used to evaluate the accuracy of the trajectories. The data also provided the monocular image corresponding to the panoramic image (see Figure 7b), which could highlight the advantages of using panoramic images in SLAM.

**Figure 10:**
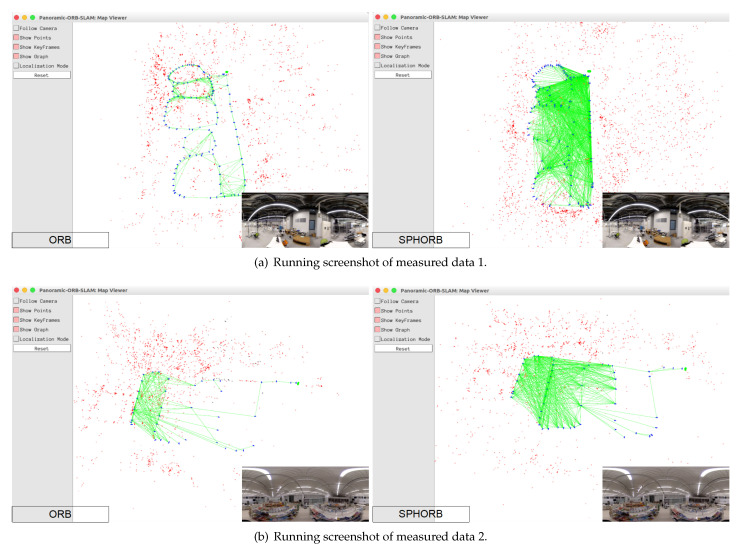
SLAM using ORB and SPHORB.

The experimental results for the first group of data are shown in Figure 10 and listed in Table 3. It can be seen from the figure that the common view of SLAM when using SPHORB is much denser than when using ORB. This is due to the fact that the number of matching points of SPHORB is higher, which makes the constraint between frames stronger and the final accuracy higher.

The experimental results for the second group of data are shown in Table 4. The two groups of InteriorNet simulation data were used to complete three groups of experiments. Panoramic images were used for the SLAM with the SPHORB and ORB algorithms, and monocular images were used for the SLAM with the ORB algorithm. Due to the violent movement in the simulated data, tracking failure occurred in the monocular images, whereas no tracking failure occurred in the SLAM experiments with the panoramic images. These comparative experiments proved the advantage of SLAM in respect of panoramic images.

In Table 4, except for “Monocular ORB”, which experimented with monocular images, the other entries all experimented with panoramic images. The results show that in the column of SPHORB, the initial effect, the average number of matches per frame, and the total number of final map points, are the best among the three groups of experiments, but its shortcomings are also very obvious, and the speed is slow.

**Figure 11:**
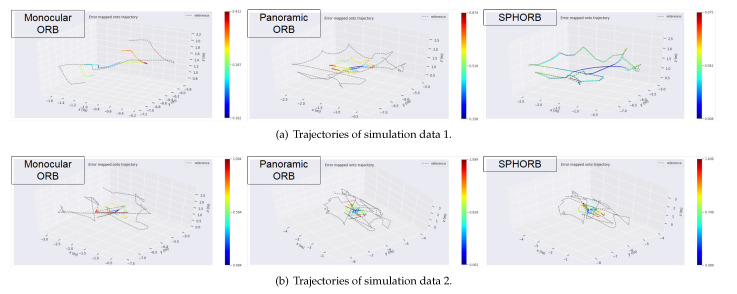
Trajectories drawn by EVO.

Table 5 and Figure 11 show the results of the evaluation with the EVO Python package [48]. The headers max, mean, min, rmse, and std in Table 5 represent the maximum, average, minimum, root mean square error, and standard deviation of the positioning error, respectively. From the experimental results for the simulation 1 data, it is clear that the rmse of SLAM with the SPHORB algorithm is the lowest. The trajectory of the SPHORB algorithm is closest to the true value of the trajectory. In contrast, the trajectory of monocular ORB is not complete, because it lost many frames, resulting in only a short tracking result.

The scene of simulation 2 data is more complex, so the three groups of experiments did not obtain good results. As shown in Table 4, the monocular ORB had tracking failures, so its results are not comparable with the other two groups. In Table 5, we put the symbol (**✗**) on the corresponding row. The accuracy of panoramic SPHORB was slightly better than that of panoramic ORB, but the time consumed by SPHORB was about four times that of ORB. It can be seen that, for the case of a complex scene, the accuracy of SPHORB does not show a great advantage over ORB, and it does take more time.

## 8. Conclusions

In this paper, we have studied the spherical imaging model and a method of panoramic visual SLAM. We have developed a SLAM positioning system suitable for panoramic vision. Through the research of this paper, the following conclusions can be drawn:

(1) For the spherical model, we compared the spherical surface to the Earth. The pixel coordinates on the sphere were expressed in latitude and longitude. The equations derived by this method are concise and easy to understand, which provides convenience for the back-end optimization part of panoramic SLAM.
(2) Experiments show that most of the time, ternary SIFT outperforms binary SIFT and SIFT in accuracy and efficiency. The precision of ternary SIFT is slightly less than SIFT only when the number of feature points is very small (i.e., less than 500), but this is acceptable.
(3) Spherical images have a higher resolution and more feature points, which has greater advantages than monocular images. However, the distortion of spherical images is serious. After weighing the relationship between accuracy and speed, it was found that the SPHORB algorithm is the most suitable among the feature extraction and matching algorithms mentioned in this paper for panoramic visual SLAM positioning systems.

## Figures and Tables

**Table 1:** Evaluation of matching results for different kinds of SIFT.

Data Type	Matching Method	Rough Matching Number	Fine Matching Number	Fine Matching Rate	Matching Time (s)
Group 1 (Indoor, More Feature Points)	SIFT	1196	475	39.72%	1.104
	binary SIFT	1212	460	37.95%	0.731
	ternary SIFT	1075	439	40.84%	0.698
Group 2 (Rotate 90 degrees)	SIFT	1397	365	26.13%	1.578
	binary SIFT	1403	354	25.23%	1.007
	ternary SIFT	1295	389	30.04%	0.979
Group 3 (Outdoor, Fewer Feature Points)	SIFT	191	92	48.17%	0.319
	binary SIFT	267	82	30.71%	0.224
	ternary SIFT	196	68	34.69%	0.214

**Table 2:** Evaluation of matching results for ORB and SPHORB.

Data Type	Matching Method	Rough Matching Number	Fine Matching Number	Fine Matching Rate	Matching Time (s)
Group 1 (Indoor, More Feature Points)	ORB	1458	978	67.08%	1.868
	SPHORB	1323	826	62.43%	1.895
Group 2 (Rotate 90 degrees)	ORB	2162	1190	55.04%	3.791
	SPHORB	4860	1208	24.86%	4.099
Group 3 (Outdoor, Fewer Feature Points)	ORB	350	166	47.43%	3.263
	SPHORB	267	165	61.80%	3.695

**Table 3:** Comprehensive evaluation of ORB and SPHORB.

Data Type	Matching Method	Initial Frame ID	Mean Matches Per Frame	Mean Time Per Frame (s)
Measured Data 1	ORB	108	191	0.102
	SPHORB	11	329	0.483
Measured Data 2	ORB	238	150	0.102
	SPHORB	204	204	0.49

**Table 4:** Comprehensive evaluation of monocular ORB, panoramic ORB and SPHORB.

Data Type	Matching Method	Initial Frame ID	Mean Matches Per Frame	Mean Time Per Frame (s)	Lost Frame IDs
Simulation Data 1	Monocular ORB	12	236	0.027	167-876
	Panoramic ORB	55	802	0.143	None
	SPHORB	4	855	0.525	None
Simulation Data 2	Monocular ORB	4	205	0.029	449-574, 692-873
	Panoramic ORB	4	327	0.12	None
	SPHORB	2	589	0.484	None

**Table 5:** EVO evaluation of monocular ORB, panoramic ORB and SPHORB. (**✗**) means there exist tracking failures.

Data Type	Matching Method	Max	Mean	Min	Rmse	Std
Simulation Data 1	Monocular ORB	**✗**	**✗**	**✗**	**✗**	**✗**
	Panoramic ORB	0.874	0.576	0.158	0.597	0.155
	SPHORB	0.075	0.035	0.008	0.036	0.011
Simulation Data 2	Monocular ORB	**✗**	**✗**	**✗**	**✗**	**✗**
	Panoramic ORB	1.589	0.868	0.062	0.937	0.354
	SPHORB	1.409	0.825	0.089	0.885	0.319

## Data Availability

The data that support the findings of this study are openly available in InteriorNet Dataset at https://interiornet.org/ and in SPHORB at https://github.com/tdsuper/SPHORB/tree/master/Image.
